# A pilot study on the identification of human papillomavirus genotypes in tongue cancer samples from a single institution in Ecuador

**DOI:** 10.1590/1414-431X20187810

**Published:** 2018-10-08

**Authors:** G.M. Delgado Ramos, T.G. Cotter, L. Flor Ramos, V. Torres Floril, G.A. Ramos Martinez, J.C. Ruiz-Cabezas

**Affiliations:** 1 School of Medicine, Universidad Católica de Santiago de Guayaquil, Guayaquil, Ecuador; 2 Department of Medicine, University of Chicago Medical Center, Chicago, IL, USA; 3 Department of Hematology and Oncology, Instituto Oncológico Nacional de la Sociedad de Lucha Contra el Cancer, Guayaquil, Ecuador; 4 Department of Molecular Biology, Instituto Oncológico Nacional de la Sociedad de Lucha Contra el Cancer, Guayaquil, Ecuador

**Keywords:** Human papillomavirus, Oropharyngeal, Oral, Ecuador, Head and neck cancer, Epidemiology

## Abstract

The relationship between human papillomavirus (HPV) and oropharyngeal squamous cell carcinoma has been established. However, data from Ecuador is limited. The objective of this study was to characterize HPV infection in Ecuadorian patients with tongue cancer. Fifty-three patients with tongue cancer treated at the tertiary referral center Sociedad de Lucha Contra el Cancer (SOLCA), Guayaquil, between 2006 and 2011 were identified. Linear Array^®^ HPV genotyping was used to identify the presence and types of HPV on formalin-fixed paraffin-embedded biopsy samples from these patients with tongue cancer. HPV was identified in 42% (n=22) and high-risk (HR) HPV in 17% (n=9), with 18 different HPV types identified. The most common types were the HR HPV 33 (14%) and low-risk HPV 67 (14%), followed by the HR HPV 58. More than one HPV type was identified in 27.3% of cases. HPV 33 was frequently associated with other HPV types. No statistically significant differences in gender (P=0.58) and age (P=0.12) were observed between HPV-positive and HPV-negative cases. HPV was identified in almost half of the tongue cancer samples, with subtypes 33 and 67 being the most common. This suggested that HPV played an important role in this disease in the population studied. Given these results, current HPV vaccines may not be as effective in reducing tongue cancer rates in this population.

## Introduction

More than 680,000 cases of head and neck cancer (HNC) are diagnosed worldwide each year, and approximately 375,000 patients die annually from this disease (1). An estimated 440,000 of HNC arise from the oral cavity and pharynx (1) and 90% are squamous cell carcinoma (SCC) (2). In Ecuador, there are up to 446 new cases of oropharyngeal cancer (OPC) annually, representing 3% of the total incidence of cancer in this country (1).

Tobacco and alcohol use are risk factors strongly associated with the development of OPC. In combination, these risk factors are thought to have a multiplicative effect, rather than just an additive effect (3). Recently, there has been a decreased incidence in certain types of HNC (oral cavity, hypo-pharyngeal, and laryngeal cancers), which has been attributed to decreased consumption of tobacco and alcohol (2). However, some subtypes such as OPC, have maintained a stable incidence over time, with even an increased incidence noted in certain regions (2,4). As an example, the overall incidence of OPC increased by 28% in a U.S. population-based study during the years 1988 and 2004 (5).

Human papillomavirus (HPV) infection has been proposed as a cause for certain HNC, particularly OPC (6,7). A concomitant rise in incidence of HPV infection has been noted with this increased incidence in OPC (2,5,8,9). HPV-associated OPC expresses the E6 and E7 proteins, which bind and inhibit the tumor suppressors p53 and pRB, respectively (6). It is more common in younger patients (2), with a higher lifetime number of vaginal and oral sex partners (7). This differs from HPV-negative OPC, which is associated with tobacco and alcohol use, poor oral hygiene (10), and usually has a large number of deletions or mutations on the tumor suppressor genes and a higher tumor EGFR expression (11,12). Oropharyngeal cancer associated with HPV is more treatment-responsive with a better overall prognosis than those not related to HPV, and thus it is important to make this differentiation at the time of diagnosis (6,11,13,14). Of all the HPV types, HPV 16 is the most commonly associated with OPC (6).

There is no data showing the impact of HPV-related OPC and the specific HPV types associated with this pathology in Ecuador. The objective of this study was to identify the presence and prevalence of HPV types in tongue cancer cells from a cohort of Ecuadorian patients, and to compare these results with HPV type prevalence in other countries.

## Material and Methods

### Patients

All patients with a diagnosis of primary tongue cancer and corresponding formalin-fixed paraffin-embedded (FFPE) tissue samples, treated at the Instituto Oncológico Nacional de la Sociedad de Lucha Contra el Cancer (ION-SOLCA), Guayaquil, Ecuador, between January 1, 2006 and December 31, 2011, were identified. Clinical characteristics of the patients (age, gender, histopathological diagnosis, tumor dimensions, tobacco use (yes or no), and alcohol use (yes or no)) were recorded after review of the patient medical records. Tumor size was classified following the American Joint Committee on Cancer (AJCC) staging manual, eighth edition (2017). Institutional Review Board approval (including the ethics committee) was obtained prior to study commencement. All data obtained was treated in accordance with the institution's patient confidentiality rules and regulations, including protection of patient names by coding of the samples.

### Tissue analysis

A microtome was used to obtain 5–10-μm thick tissue slices of the FFPE tissue blocks, which were then placed in separate encoded tubes and stored. Contamination was prevented by discarding the first 5–8 slices of each FFPE tissue block, using different blades, and discarding gloves and cleaning the material with 70% ethanol after each case had been sliced. Genetic material was obtained through the QIAmp isolation method, using the protocol described in previous published studies (15,16).

HPV genetic material and genotypes were identified using the Linear Array^®^ HPV genotyping test (Roche Molecular Diagnostics, Germany) according to the recommendations of the manufacturer. This test combines polymerase chain reaction (PCR) amplification of target DNA and reverse line-blot hybridization for the identification of thirty-seven anogenital HPV DNA genotypes, including 14 high-risk (HR) HPV types (HPV 16, 18, 31, 33, 35, 39, 45, 51, 52, 56, 58, 59, 66 and 68) and 23 low-risk (LR) HPV types (HPV 6, 11, 26, 40, 42, 53, 54, 55, 61, 62, 64, 67, 69, 70, 71, 72, 73, 81, 82, 83, 84, IS39, and CP6108). It contains an internal control (a gene probe corresponding to the human β-globin gene), that determines the presence of sufficient cellular material in the biological samples. A result is considered valid when the bands of the internal β-globin control are positive. All of the cases in this study were valid. Hybridization and HPV genotyping were performed as described by the manufacturer (Roche Molecular System, Inc.), HPV types were identified by comparing the hybridization signals with the Linear Array HPV reference guide. Both positive and negative controls (including HPV 16, 18, 31) were used and adequately typed in order to validate the genotyping test.

### Data management

Programs Numbers and Excel were used for data entry and management. Comparison between groups was performed using Fisher’s exact test. The level of significance was P<0.05. Continuous data are reported as means±SD. Categorical data are reported as proportions and percentages. IBM SPSS Statistics version 24 (IBM, USA) was used for statistical analyses.

## Results

The cohort consisted of 53 patients with tongue cancer. Mean age was 61.8±17.3, 29 patients (55%) were male, 49 patients (92%) had SCC and 22 patients (42%) were positive for HPV. Demographic and clinical characteristics of the cohort are shown in Table 1.

**Table 1:** Demographics and clinical characteristics of SOLCA-Guayaquil tongue cancer cohort (2006-2011) (n=53).

	HPV + (n=22)	HPV - (n=31)
Age, mean (SD)	57.4 (16.7)	64.9 (17.3)
Male, n (%)	11 (50)	18 (58)
Tobacco use		
Yes, n (%)	0 (0)	8 (26)
No, n (%)	18 (82)	18 (58)
Unknown, n (%)	4 (18)	5 (16)
Alcohol use		
Yes, n (%)	4 (18)	5 (16)
No, n (%)	15 (68)	20 (65)
Unknown, n (%)	3 (14)	6 (19)
Diagnosis		
SCC, n (%)	21 (95)	29 (94)
Other, n (%)	1 (5)	2 (6)
Adenocarcinoma, n (%)	1 (5)	1(3)
Mucoepidermoid carcinoma, n (%)	0	1(3)

SOLCA: Sociedad de Lucha contra el Cancer; HPV: human papillomavirus; +: positive; –: negative; n: number; SD: standard deviation; SCC: squamous cell carcinoma.

Of the 22 cases in which HPV genome was detected, 11 were female (46% of total female cases) while 11 were male (38% of total male cases); no statistically significant difference was found between genders (P=0.58). The mean age for the patients that had HPV genome was 57 years with a standard deviation of 16.7; no statistically significant difference was found between the ages of HPV positive and negative cases (t_51_= –1.581, P=0.12, 95% CI [–17, 2]). There were no smokers in the HPV-positive patients, compared with 8 smokers in the HPV-negative patients (P=0.029). Four patients reported alcohol intake in the HPV-positive cohort compared with 5 patients in the HPV-negative cohort (P=0.92). Among the HPV-positive cohort, the anterior two-thirds of the tongue was the most common site of cancer (n=9, 41%), followed by the base of the tongue (n=5, 23%), with 95% having a histopathological diagnosis of SCC. The most common tumor stages were T2 (n=9, 41%) and T4 (n=6, 27%) (Supplementary Table S1).

**Figure 1:**
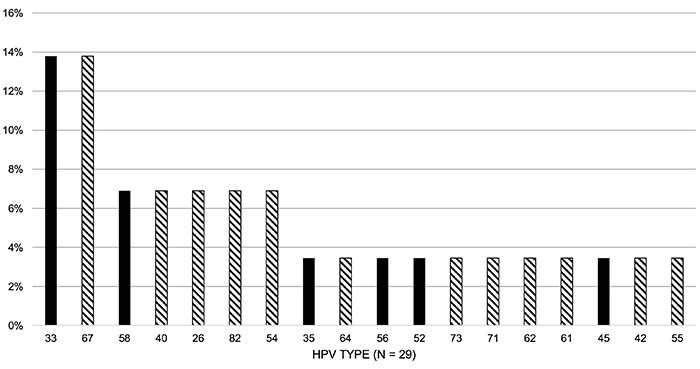
Human papillomavirus type prevalence of SOLCA-Guayaquil tongue cancer cohort (2006–2011). HPV: human papillomavirus; Solid black color: high-risk HPV; Diagonal pattern: low risk HPV. SOLCA: Sociedad de Lucha contra el Cancer.

A total of 18 different types of high- and low-risk HPV were identified, among which HPV 33 (HR) and 67 (LR) were the most common, being found in 14% (n=4) each (Figure 1). More than one HPV type was found in 27% (n=6) of the cases (there was one case with each HPV combination: 35–40, 58–64, 33–56, 33–62, 26–33, 52–73–82). HPV 33 was frequently associated with other HPV types.

## Discussion

In a cohort of Ecuadorian patients with tongue cancer, HPV (both HR and LR) was found in almost half of the cancers. There was a higher prevalence of HPV in younger patients compared with older patients. Although this difference was not statistically significant, it may have approached significance with a larger sample size. Tobacco consumption was significantly higher in HPV-negative patients, consistent with prior studies where an inverse correlation between smoking and HPV has been established (11). There was no significant difference between alcohol consumption or between genders among the HPV-positive and HPV-negative groups.

A number of Ecuadorian studies identifying HPV types have been performed in anogenital cancers, but not in HNC. The majority of these studies show HPV 16 as the most prevalent type (17–21). In this cohort, the most prevalent HPV types detected in tongue tumors were HPV 33 (HR) and 67 (LR). High-risk HPV 58, 35, 56, 52, and 45 were also detected. Notably, we did not identify any HPV 16 or 18, which is in contrast to other international studies that show HPV 16 as the most commonly associated with HNC (2,6,8,22,23). A Swedish study identified HPV 16 in 85% of tongue cancer samples, while HPV 33 was found in 10% (22). A similar trend was identified in an international study involving countries from Europe, Asia, Africa, and the USA (8). A more recent USA study found significant associations between HPV types 16, 18, 33, and 52 and HNC (23). The absence of HPV 16 in this cohort may be indicative of a different pattern of HPV types in the Ecuadorian tongue cancer cohort; however, a larger study is required to confirm this.

Two HPV vaccines are currently commercially available in Ecuador - bivalent Cervarix^®^ (HPV 16 and 18), which is recommended at age 9 years and has been available since 2014 in the national immunization program (24,25), and tetravalent Gardasil^®^ (HPV 6, 11, 16, and 18). These vaccines prevent HPV infection and associated anogenital cancer (both) and anogenital warts (Gardasil^®^) (26). It has been shown that prophylactic HPV vaccination reduces the rates of oral HPV 16 and 18 infections (27,28), which in theory may ultimately reduce HNC rates. Of concern in this population is the absence of HPV type 16 or 18 in the tongue cancer samples. Thus, the currently available vaccines may not be as effective in reducing HNC rates in Ecuador. Despite this, it is important to note that HPV vaccines have been shown to provide cross-protection against other HPV strains such as types 31, 33, and 45; however, this cross-protection may wane over time (29,30). The recently introduced nonavalent vaccine Gardasil^®^ (HPV 6, 11, 16, 18, 31, 33, 45, 52, and 58) in theory may be more effective in reducing tongue cancer (and other HPV-associated HNC) rates given the prevalence of HPV 33, 45, 52, and 58 in this cohort, however, it is cost-prohibitive for the vast majority of the Ecuadorian population.

This study had limitations, including its retrospective nature, which resulted in some missing data regarding alcohol and tobacco use, and precise cancer location. This may have introduced bias to the results; however, the number of missing data was low and thus may not have affected the overall results. The sample size was low despite involving all patients with a diagnosis of tongue cancer with available cancer tissue for 5 consecutive years. Given the time elapsed between initial specimen acquisition and the HPV analyses in FFPE material, there may have been partially degraded DNA at the time of testing. Moreover, while HPV presence was determined in the samples, their biological activity (by identifying the presence of p16, or of E6 and E7 mRNA) was not assessed in this sample.

In conclusion, this pilot study identified HPV genome in a high percentage of tongue cancer tumors, further supporting the hypothesis that HPV has an important role in this disease. In this Ecuadorian tongue cancer cohort, HPV 16 and 18 were not present, which may have implications for the current HPV vaccination program should HNC be considered in the future. Larger studies are required to confirm if this HPV type pattern is representative of the Ecuadorian population. Furthermore, identification of the presence of p16 would be of particular interest when identifying the prevalence of HPV infection.

## Supplementary Material

Click here to view [pdf].

## References

[1] GLOBOCAN 2012 v1.0, Cancer Incidence and Mortality Worldwide: IARC CancerBase No (2013). International Agency for Research on Cancer.

[2] Joseph AW, D'Souza G (2012). Epidemiology of human papillomavirus-related head and neck cancer. Otolaryngol Clin North Am.

[3] Ryerson AB, Peters ES, Coughlin SS, Chen VW, Gillison ML, Reichman ME (2008). Burden of potentially human papillomavirus-associated cancers of the oropharynx and oral cavity in the US, 1998-2003. Cancer.

[4] Chaturvedi AK, Anderson WF, Lortet-Tieulent J, Curado MP, Ferlay J, Franceschi S (2013). Worldwide trends in incidence rates for oral cavity and oropharyngeal cancers. J Clin Oncol.

[5] Chaturvedi AK, Engels EA, Pfeiffer RM, Hernandez BY, Xiao W, Kim E (2011). Human papillomavirus and rising oropharyngeal cancer incidence in the United States. J Clin Oncol.

[6] Herrero R, Castellsague X, Pawlita M, Lissowska J, Kee F, Balaram P (2003). Human papillomavirus and oral cancer: the International Agency for Research on Cancer Multicenter Study. J Natl Cancer Inst.

[7] D'Souza G, Kreimer AR, Viscidi R, Pawlita M, Fakhry C, Koch WM (2007). Case-control study of human papillomavirus and oropharyngeal cancer. N Engl J Med.

[8] Castellsague X, Alemany L, Quer M, Halec G, Quiros B, Tous S (2016). HPV involvement in head and neck cancers: comprehensive assessment of biomarkers in 3680 patients. J Natl Cancer Inst.

[9] Mourad M, Jetmore T, Jategaonkar AA, Moubayed S, Moshier E, Urken ML (2017). Epidemiological trends of head and neck cancer in the United States: A SEER population study. J Oral Maxillofac Surg.

[10] Gillison ML, D'Souza G, Westra W, Sugar E, Xiao W, Begum S (2008). Distinct risk factor profiles for human papillomavirus type 16-positive and human papillomavirus type 16-negative head and neck cancers. J Natl Cancer Inst.

[11] Kumar B, Cordell KG, Lee JS, Worden FP, Prince ME, Tran HH (2008). EGFR, p16, HPV Titer, Bcl-xL and p53, sex, and smoking as indicators of response to therapy and survival in oropharyngeal cancer. J Clin Oncol.

[12] Rampias T, Sasaki C, Psyrri A (2014). Molecular mechanisms of HPV induced carcinogenesis in head and neck. Oral Oncol.

[13] Ramqvist T, Dalianis T (2011). An epidemic of oropharyngeal squamous cell carcinoma (OSCC) due to human papillomavirus (HPV) infection and aspects of treatment and prevention. Anticancer Res.

[14] Saba NF, Goodman M, Ward K, Flowers C, Ramalingam S, Owonikoko T (2011). Gender and ethnic disparities in incidence and survival of squamous cell carcinoma of the oral tongue, base of tongue, and tonsils: a surveillance, epidemiology and end results program-based analysis. Oncology.

[15] Sam SS, Lebel KA, Bissaillon CL, Tafe LJ, Tsongalis GJ, Lefferts JA (2012). Automation of genomic DNA isolation from formalin-fixed, paraffin-embedded tissues. Pathol Res Pract.

[16] Kocjan BJ, Maver PJ, Hosnjak L, Zidar N, Odar K, Gale N (2012). Comparative evaluation of the abbott realtime high risk HPV test and INNO-LiPA HPV genotyping extra test for detecting and identifying human papillomaviruses in archival tissue specimens of head and neck cancers. Acta Dermatovenerol Alp Pannonica Adriat.

[17] Brown CR, Leon ML, Munoz K, Fagioni A, Amador LG, Frain B (2009). Human papillomavirus infection and its association with cervical dysplasia in Ecuadorian women attending a private cancer screening clinic. Braz J Med Biol Res.

[B18] Dalgo Aguilar P, Lojan Gonzalez C, Cordova Rodriguez A, Acurio Paez K, Arevalo AP, Bobokova J (2017). Prevalence of high-risk genotypes of human papillomavirus: women diagnosed with premalignant and malignant pap smear tests in southern Ecuador. Infect Dis Obstet Gynecol.

[B19] Garcia Muentes GD, Garcia Rodriguez LK, Burgos Galarraga RI, Almeida Carpio F, Ruiz Cabezas JC (2016). Genotypes distribution of human papillomavirus in cervical samples of Ecuadorian women. Rev Bras Epidemiol.

[B20] Marengo C, Rivadeneira N, Ruiz-Cabezas JC (2010). Tipificación de HPV en cáncer de cérvix en inclusión de parafina. Realizado en ION SOLCA [in Spanish]. Rev Oncol.

[21] Tornesello ML, Buonaguro L, Izzo S, Lopez G, Vega X, Maldonado Reyes CF (2008). A pilot study on the distribution of human papillomavirus genotypes and HPV-16 variants in cervical neoplastic lesions from Ecuadorian women. J Med Virol.

[22] Attner P, Du J, Nasman A, Hammarstedt L, Ramqvist T, Lindholm J (2010). The role of human papillomavirus in the increased incidence of base of tongue cancer. Int J Cancer.

[23] Michaud DS, Langevin SM, Eliot M, Nelson HH, Pawlita M, McClean MD (2014). High-risk HPV types and head and neck cancer. Int J Cancer.

[24] Ministerio de la Salud Pública del Ecuador, Secretaría Nacional de Planifcación y Desarrollo, Organización Panamericana de la Salud / Organización Mundial de la Salud (OPS/OMS), Representación en Ecuador de la Organización Panamericana de la Salud (2017). Evaluación de la Estrategia Nacional de Inmunizaciones Ecuador 2017.

[25] Subsecretaría Nacional de Vigilancia de la Salud Pública, Dirección Nacional de Estrategias de Prevención y Control, Inmunizaciones (2015). ENd. Esquema de Vacunación Familiar, Ecuador 2015.

[26] Schiller JT, Castellsague X, Garland SM (2012). A review of clinical trials of human papillomavirus prophylactic vaccines. Vaccine.

[27] Herrero R, Quint W, Hildesheim A, Gonzalez P, Struijk L, Katki HA (2013). Reduced prevalence of oral human papillomavirus (HPV) 4 years after bivalent HPV vaccination in a randomized clinical trial in Costa Rica. PLoS One.

[28] Chaturvedi AK, Graubard BI, Broutian T, Pickard RKL, Tong ZY, Xiao W (2018). Effect of Prophylactic Human Papillomavirus (HPV) Vaccination on Oral HPV Infections Among Young Adults in the United States. J Clin Oncol.

[29] GlaxoSmithKline Vaccine HPV-007 Study Group, Romanowski B, de Borba PC, Naud PS, Roteli-Martins CM, De Carvalho NS, et al (2009). Sustained efficacy and immunogenicity of the human papillomavirus (HPV)-16/18 AS04-adjuvanted vaccine: analysis of a randomised placebo-controlled trial up to 6.4 years. Lancet.

[30] Malagon T, Drolet M, Boily MC, Franco EL, Jit M, Brisson J (2012). Cross-protective efficacy of two human papillomavirus vaccines: a systematic review and meta-analysis. Lancet Infect Dis.

